# Trajectories of glycated hemoglobin of T2DM and progress of arterial stiffness: a prospective study

**DOI:** 10.1186/s13098-023-01108-8

**Published:** 2023-06-23

**Authors:** Kun Li, Bin Cao, Huan Dong, Longyan Yang, Dong Zhao

**Affiliations:** 1 Center for Endocrine Metabolism and Immune Diseases, Beijing Luhe Hospital, Capital Medical University, Beijing, 101149 China; 2 Beijing Key Laboratory of Diabetes Research and Care, Beijing, 101149 China

**Keywords:** Diabetes, HbA1c, Arterial stiffness, Analysis of trajectory

## Abstract

**Aim:**

This study aimed to describe the different trajectories groups of HbA1c during the long-term treatment of diabetes and explore the effect of glycemic control on the progression of arterial stiffness.

**Method:**

The study participants registered at the National Metabolic Management Center (MMC) of Beijing Luhe hospital. The latent class mixture model (LCMM) was used to identify distinct trajectories of HbA1c. We calculated the change value of baPWV (ΔbaPWV) of each participant between the whole follow-up time as the primary outcome. Then we examined the associations between each HbA1c trajectory pattern and ΔbaPWV using covariate-adjusted means (SE) of ΔbaPWV, which were calculated by multiple linear regression analyses adjusted for the covariates.

**Results:**

After data cleaning, a total of 940 type 2 diabetes patients aged 20–80 years were included in this study. According to the BIC, we identified four discrete trajectories of HbA1c: Low-stable, U-shape, Moderate-decrease, High-increase, respectively. Compared with the low-stable group of HbA1c, the adjusted mean values of baPWV were significantly higher in U-shape, Moderate-decrease, and High-increase groups (all P < 0.05, and P for trend < 0.001), the mean values (SE) were 82.73 (0.08), 91.19 (0.96), 116.00 (0.81) and 223.19 (11.54), respectively.

**Conclusion:**

We found four different trajectories groups of HbA1c during the long-term treatment of diabetes. In addition, the result proves the causal relationship between long-term glycemic control and arterial stiffness on a time scale.

**Supplementary Information:**

The online version contains supplementary material available at 10.1186/s13098-023-01108-8.

## Introduction

Type 2 diabetes mellitus (T2DM) is a significant health problem, especially in low and middle-income countries [1]. The new epidemiological investigation of IDF showed that 537 million people had diabetes in 2021 [2]. Diabetic vascular complications are the leading cause of crippling and death of diabetic patients and seriously affect patients’ quality of life, including diabetic nephropathy (DN), and cardiovascular (CVD), et al. [3–5].

Microvascular damage from significant artery stiffness (LAS) may affect glucose homeostasis [6]. Moreover, the recent mendelian randomization studies demonstrated a two-way causal relationship between glucose homeostasis and arterial stiffness [7]. Therefore, the interaction effect of diabetes and arterial stiffness increases the risk of CVD.

Brachial-ankle pulse wave velocity (baPWV) has been widely used to indicate arterial stiffness in a clinical setting. In extensive population studies, it is the risk factor for CVD and Chronic kidney disease (CKD) [8–10]. Some cross-sectional studies present the association between baPWV and HbA1c [11]. However, most of these studies could not describe long-term glycemic control’s effect on the progression of vascular stiffness.

Therefore, this study aimed to descript the different trajectories groups of HbA1c during the long-term treatment of diabetes and explore the effect of long-term glycemic control on the progression of vascular stiffness using the trajectory model analysis method. The participants registered at the National Metabolic Management Center (MMC), an innovation project for the management of metabolic diseases and complications in China [12]. We hypothesize that the different trajectories of HbA1c may influence the progress of vascular stiffness, which could prove the causal relationship between diabetes and arterial stiffness on a time scale and has clinical significance for treating diabetes.

## Method

### Study design and population

All participants were diagnosed with type 2 diabetes mellitus in this study and registered at the MMC of Beijing Luhe hospital from June 2017 to October 2022. The MMC is a national project to manage metabolic patients according to the same standard. All the participants accepted blood sample collection, systematic physical examination, and oral questionnaire interviews. The protocol of this project was published previously [12]. T2DM was diagnosed according to the 1999 World Organization criteria if they had a fasting plasma glucose ≥ 7.0 mmol/L or 2-h plasma glucose ≥ 11.1 mmol/L or a self-reported physician diagnosis.

Considering the minimum age of participants in the MMC program and the life expectancy of older participants, participants aged 20–80 years has been identified as the potential research objects in the present study.

Participants were excluded according to the following criteria: [1] pregnant or nursing women; [2] malignant tumor; [3] acute complications of diabetes; [4] visited times less than three times and the following time less than 18 months; [5] missing data of critical variables.

The Medical Ethics Committee of Beijing Luhe Hospital, Capital Medical University approved the study protocol. This study was performed by the Declaration of Helsinki, and all participants provided written informed consent.

### Measurement of glycated hemoglobin and baPWV

Glycated hemoglobin (HbA1c) was the important prognostic indicator in the MMC program. The interval time between HbA1c examination for each participant was less than 6 months. Blood samples were obtained in the morning after fasting for at least 8 h. Venipuncture was performed in the median cubital vein. HbA1c levels were assayed using high-performance liquid chromatography (HPLC) with a D10 set (Bio-RAD, Hercules, CA, USA).

BaPWV was measured non-invasively by an automated recording apparatus with participants in the supine position after at least 5 min of rest (BP-203RPE III, form PWV/ABI, Omron Healthcare Co.). In this study, baPWV was calculated as La − Lb/ΔT (La and Lb are the distance from the heart to the ankle and the distance from the heart to the brachium, respectively, ΔT is the time between the wavefront of the brachial waveform and that of the ankle waveform).

### Covariates

Data were collected by trained personnel according to the protocol. The questionnaire containing information on demographic characteristics, lifestyle factors (including alcohol drinking and cigarette smoking et al.), and medical history were administered by trained interviewers. For the participants who smoked daily or almost daily, smoking status was defined as ‘yes.’ And for the participants who drank weekly or nearly weekly, their drinking status was described as ‘yes.’ Education attainment was categorized as less than high school and high school or more.

Height and body weight were measured with a standard protocol, and body mass index (BMI) was calculated as weight divided by height squared. LDL cholesterol, HDL cholesterol, and triglyceride were measured using an auto-biochemical analyzer (Roche COBAS C501; Roche Diagnostics Corporation, Germany).

### Statistical analysis

Data are described as mean ± standard deviation (SD) or median [interquartile range (IQR)] for continuous variables and as the frequency (%) for categorical variables. When data were tested as non-normal distribution, logarithmically transformed were required before statistical analysis. P values for trend were calculated using the Cochran–Armitage trend test and linear regression analyses for categorical and continuous variables across the three groups, respectively. The generalized additive models (GAMs) were used to investigate the age-dependent trend of baPWV with sex groups [13].

Multiple linear regressions were used to explore the association between HbA1c and baPWV; three models were established and adjusted for a different covariate. Model 1 was adjusted for age, sex and duration, which was a relative cruded model used to explore the based relationshiap between HbA1c and baPWV; The unhealthy lifestyle like smoke, dirnk were adverse factor of cardiovascular disease [14], so we adjuested for smoke, education, and drink plus covariate of model 1 in model 2; the model 3 was input all variables in model 2 plus BMI SBP, TC, TG, HDL, LDL, hypertension to adjust for common possible confounding factors.

The latent class mixture model (LCMM) was used to identify distinct subgroups, which may present similar trajectories of HbA1c [15–17]. The LCMM was a trajectories analysis method that applied finite mixture modeling to map the continuous variable over time or age. The *lcmm* package of R was used to execute the procedure [16]. The number of latent categories is set to 2 to 5. The optimal model was selected by the maximum Bayesian information criterion (BIC). The trajectories groups were named based on baseline HbA1c levels and the visual change patterns of HbA1c over time. A posteriori prediction probability of the lipid track group to which each participant belonged was calculated and participants were assigned to the track with the most significant posterior probability.

We calculate the change value of baPWV (ΔbaPWV) of each participant between the whole following time as the primary outcome of the follow-up. We examined the associations between each HbA1c trajectory pattern and ΔbaPWV using covariate-adjusted means (SE) of ΔbaPWV, which were calculated by multiple linear regression analyses adjusted for the covariates mentioned previously.

We did a sensitivity analysis after excluding participants taking lipid-lowering medications to assess the stability of the findings. Moreover, we also performed sex subgroup analyses to investigate the consistency of results, multiple linear regressions adjusted all of the covariables in model 3.

All statistical analyses were performed using R software (version 4.1.2, https://www.r-project.org/).

## Results

### Demographic characteristics

There were 6 108 T2DM participants registered at MMC from June 2017 to October 2022. The data cleaning was performed to detect missing values and exclude the participants who did not get at least 3rd visit or age not between 20 and 80 years. The data cleaning procedure can be seen in Additional file 1: Fig. S1. Of 940 participants, the mean (SD) age is 51.36 (11.93) at the first visit. Participants comprised 543 (57.77%) men and 397 (42.23%) women. The follow-up median time was 37.6 months. More demographic characteristics of participants are shown in Table 1.

**Fig. 1:**
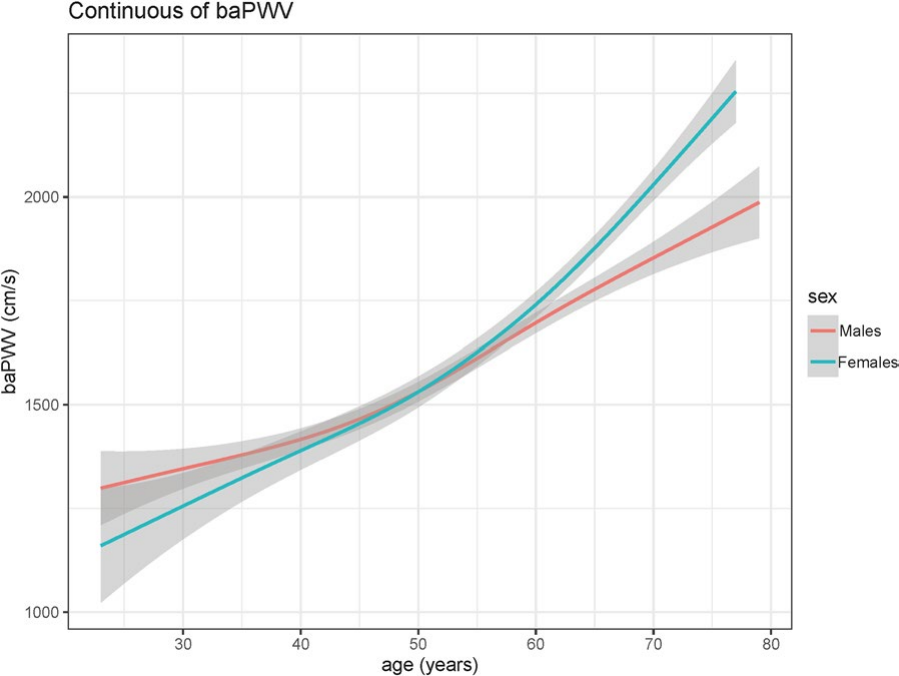
The growth curve of baPWV between 20 and 80 years in T2DM. The curve was fitted by the gamlss method

**Table 1:** Demographic characteristics of participants for the first visit of this study

	Total	Males	Females	P
N	940	543	397	
Duration, mean (SD), month	88.83 (81.48)	79.00 (78.55)	102.59 (83.61)	< 0.001
Age, mean (SD), year	51.36 (11.93)	48.97 (11.56)	54.63 (11.66)	< 0.001
Smoke, n (%)	259 (27.82)	248 (46.01)	11 (2.81)	< 0.001
Drink, n (%)	385 (41.40)	350 (64.94)	35 (8.95)	< 0.001
Hypertension, n (%)	347 (38.77)	188 (36.08)	159 (42.51)	0.600
SBP, mean (SD), mmHg	131.71 (17.22)	131.54 (17.18)	131.96 (17.29)	0.713
High school or more, n (%)	549 (58.40)	351 (64.64)	198 (49.87)	< 0.001
HbA1c, mean (SD),	8.70 (2.18)	8.73 (2.22)	8.66 (2.12)	0.615
Lipid-lowering medications, n (%)	248 (27.71)	144 (27.69)	104 (27.73)	1.000
baPWV, mean (SD), cm/s	1548.87 (305.48)	1514.69 (277.06)	1595.61 (335.30)	< 0.001
HDL, mean (SD), mmol/L	1.18 (0.28)	1.11 (0.26)	1.29 (0.29)	< 0.001
LDL, mean (SD), mmol/L	3.03 (0.91)	3.01 (0.89)	3.05 (0.93)	0.467
TC, mean (SD), mmol/L	4.77 (1.18)	4.70 (1.19)	4.87 (1.16)	0.029
TG, mean (SD), mmol/L	2.13 (2.21)	2.35 (2.43)	1.84 (1.84)	0.001
Glu, mean (SD), mmol/L	9.62 (4.30)	9.78 (4.27)	9.39 (4.33)	0.164
Weight, mean (SD), kg	74.04 (14.18)	80.10 (12.98)	65.74 (11.26)	< 0.001
BMI, mean (SD), kg/m^2^	26.65 (3.94)	27.07 (3.84)	26.08 (4.01)	< 0.001
Height, mean (SD), m	166.30 (8.77)	171.90 (6.16)	158.63 (5.33)	< 0.001

*SBP* Systolic blood pressure; *HbA1c* Glycosylated hemoglobin; *baPWV* Brachial-ankle pulse wave velocity; *HDL* High density lipoprotein; *LDL* Low density lipoprotein; *TC* total cholesterol; *TG* Triglyceride; *Glu* Fasting blood glucose; *BMI* Body mass index

### Association between baPWV and glycated hemoglobin

Growth curves of baPWV of T2DM patients by sex are shown in Fig. 1. BaPWV presents a continuously increasing trend in both males and females. Due to the different slopes of the baPWV growth curves for males and females, there is an intersection between the curves at age 53. We also represented the density of the data points by color chromaticity using the plotSimpleGamlss function of R. The results demonstrated that females were more distributed across age groups than males, which suggested more participants of male were concentrated among younger age groups (Additional file 1: Fig. S2). And the values of baPWV were more dispersed in older participants.

The association between baPWV and HbA1c was described by the value of β of multiple linear regressions (Table 2). In the crude model (model 1), the β values for a 1-SD increase of HbA1c levels were 31.27 (95% CI 14.64–47.91, P < 0.001). And after adjusting for age, sex, duration of diabetes, smoking, education, BMI, SBP, TC, TG, HDL, LDL and hypertension (model 3), HbA1c were significantly and positively associated with baPWV, and the β values for a 1-SD increase of HbA1c levels were 22.10 (95 %CI 3.55–40.66, P = 0.020).

**Table 2:** The association between HbA1c and baPWV

	β	95% CI	P
Model 1	31.27	(14.64–47.91)	< 0.001
Model 2	28.59	(11.66–45.52)	< 0.001
Model 3	22.10	(3.55–40.66)	0.020

Model 1 was adjusted for age, sex and duration; model 2 was adjusted for smoking, education, and drinking plus covariates in model 1; model 3 was adjusted for BMI SBP, TC, TG, HDL, LDL, hypertension plus covariates in model 2

### Trajectories of glycated hemoglobin

**Fig. 2:**
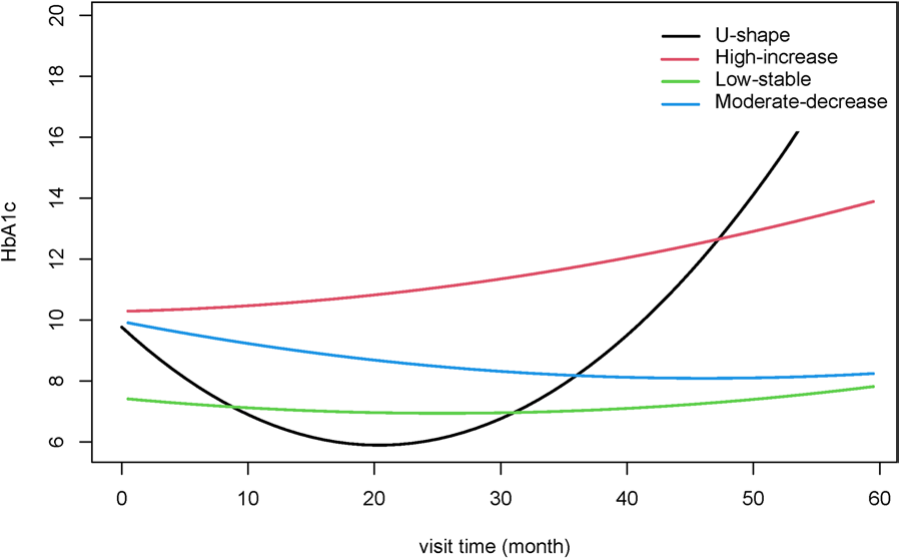
HbA1c trajectory groups during the following time. Low-stable, characterized by maintaining low HbA1c levels throughout follow-up; U-shape, the HbA1c was decreased first and reached the bottom at around two years, then the HbA1c increased during the next following time; Moderate-decrease, starting with an average HbA1c level and experiencing a slight decrease; High-increase, beginning with an elevated HbA1c level and experiencing a slow increase trend during the following time

Using the LCMM method, we identified four discrete trajectories of HbA1c during the following time (Fig. 2) according to the BIC (The selected model’s BIC was 22294.04). The trajectory groups were named based on baseline HbA1c levels and the visual change patterns of HbA1c over time: [1] low-stable, characterized by maintaining low HbA1c levels throughout follow-up; [2] U-shape, the HbA1c was decreased first and reach the bottom at around two years, then the HbA1c increased during the nest follow-up time; [3] moderate–decrease, starting with a moderate HbA1c level and experiencing a slight decrease; [4] high and increase, starting with a high HbA1c level and experiencing a slow increase trend during the following time.

Table 3 shows the demographic characteristics of each HbA1c trajectory group at the last visit. Compared to the participants in the first trajectory group of HbA1c, those in the 2nd to 4th trajectory group (U-shape, Moderate-decrease, High-increase, respectively) of HbA1c were younger with a higher level of HbA1c, fasting blood glucose, TG, and LDL (all P < 0.05).

**Table 3:** Demographic characteristics of participants in each trajectory group at the last visit

	Total	Low-stable	U-shape	Moderate- decrease	High-increase	P
N	940	723	88	98	31	
Age, mean (SD), year	54.01 (11.98)	55.07 (11.44)	47.51 (13.02)	53.10 (12.21)	50.45 (14.28)	< 0.001
Smoke, n (%)	194 (25.01)	132 (22.26)	28 (40.00)	27 (32.53)	7 (25.93)	0.017
Drink, n (%)	260 (33.64)	200 (33.73)	29 (41.43)	26 (31.33)	5 (18.52)	0.322
High school or more, n (%)	549 (58.40)	422 (58.37)	51 (57.96)	56 (57.14)	20 (64.52)	0.817
HbA1c, mean (SD)	7.53 (1.59)	7.20 (1.16)	7.22 (1.77)	8.81 (1.27)	12.11 (1.79)	< 0.001
baPWV, mean (SD), cm/s	1640.48 (379.55)	1637.86 (356.60)	1537.65 (293.56)	1695.13 (470.96)	1820.94 (637.78)	0.049
HDL, mean(SD), mmol/L	1.25 (0.32)	1.27 (0.31)	1.17 (0.29)	1.25 (0.32)	1.24 (0.50)	0.191
LDL, mean (SD), mmol/L	2.81 (0.85)	2.77 (0.81)	2.87 (0.80)	2.83 (0.91)	3.53 (1.33)	< 0.001
TC, mean (SD), mmol/L	4.63 (1.23)	4.57 (1.10)	4.59 (1.08)	4.69 (1.35)	6.01 (2.59)	< 0.001
TG, mean (SD), mmol/L	2.09 (3.68)	1.90 (2.25)	2.21 (3.52)	2.31 (3.65)	5.29 (14.41)	< 0.001
Glu, mean (SD), mmol/L	8.56 (3.18)	8.08 (2.43)	8.27 (3.48)	11.01 (4.44)	12.75 (5.58)	< 0.001
Weight, mean (SD), kg	73.19 (14.15)	72.88 (13.94)	77.30 (16.37)	72.28 (13.20)	71.76 (14.16)	0.918
BMI, mean (SD), kg/m^2^	26.45 (3.84)	26.45 (3.88)	26.74 (4.10)	26.33 (3.54)	25.92 (3.05)	0.642
Height, mean (SD), m	165.92 (9.17)	165.58 (9.11)	169.49 (8.68)	165.32 (9.39)	165.67 (9.43)	0.502

### Glycated hemoglobin trajectory groups and baPWV

**Fig. 3:**
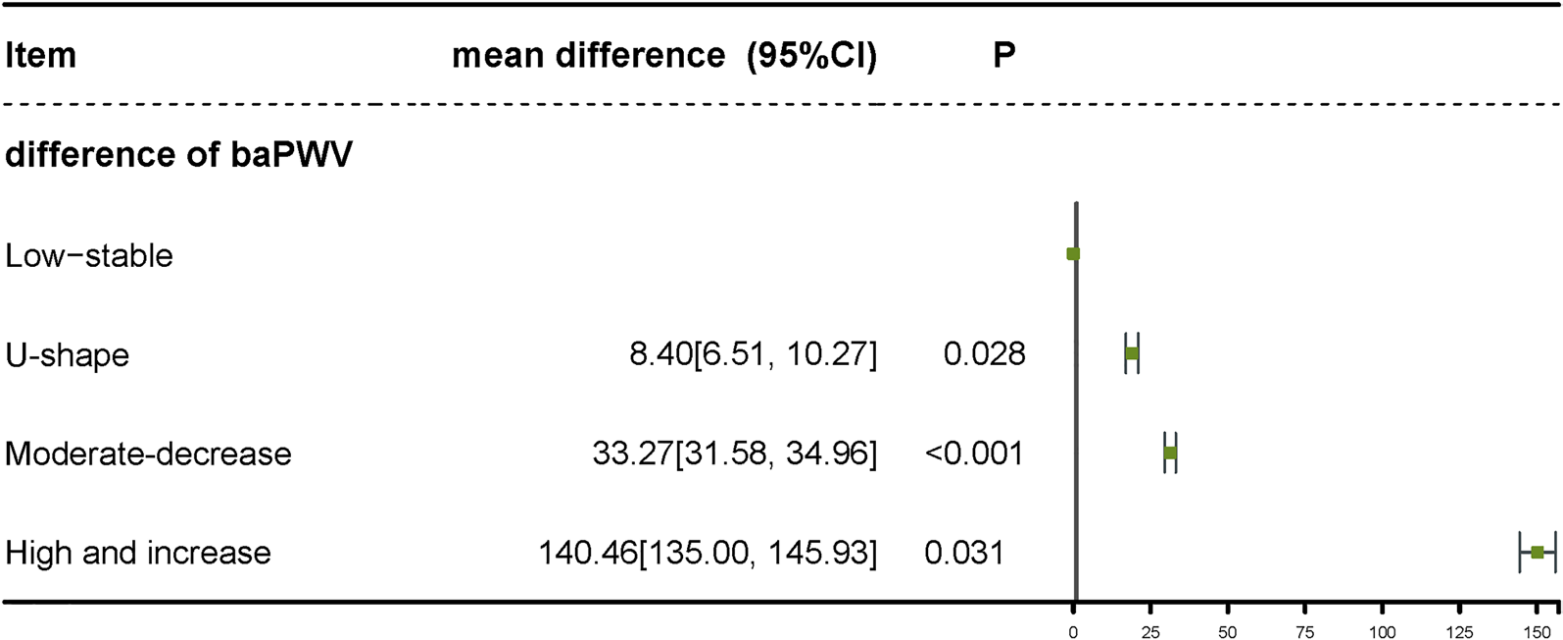
Mean differences of the change value of baPWV among HbA1c trajectory groups. The model was adjusted for age, sex, duration of diabetes, smoking, education, BMI, SBP, TC, TG, HDL, LDL and hypertension

As shown in Table 4, compared with the low-stable group of HbA1c, the adjusted mean values of ΔbaPWV were significantly higher in U-shape, Moderate-decrease (all P < 0.05, and P for trend < 0.001), and High-increase group, the mean values (SE) were 82.73 (0.08), 91.19 (0.96), 116.00 (0.81) and 223.19 (11.54), respectively. Figure 3 presents the result of the baPWV change value of the difference in groups, consistent with Table 4.

**Table 4:** Covariate-adjusted means of change of bapwv by HbA1c Trajectory Group

Trajectory group	Participants, n (%)	Change of baPWV, mean (SE), cm/s	P value	P for Trend
HbA1c				< 0.001
Low-stable	723 (76.91)	82.73 (0.08)	NA	
U-shape	88 (9.36)	91.13 (0.96)	0.028	
Moderate-decrease	98( 10.43)	116.00 (0.81)	< 0.001	
High-increase	31 (3.30)	223.19 (11.54)	0.031	

Model was adjusted for age, sex, duration of diabetes, smoking, during, education, BMI, SBP, TC, TG, HDL, LDL and hypertension

### Sensitivity analysis

To assess the stability of the findings, we did a sensitivity analysis after excluding participants taking lipid-lowering medications (Table 5). The adjusted mean values of ΔbaPWV remained significantly higher in the U shape and High-increase groups (P < 0.05), the P for trend was 0.017.

**Table 5:** Covariate-adjusted means of change of bapwv in participants without taking lipid-lowering medication by HbA1c Trajectory Group (sensitivity analysis)

Trajectory group	Participants, n (%)	Change of baPWV, mean (SE), cm/s	P value	P for trend
Low-stable	487 (75.27)	71.05 (0.14)	NA	0.017
U-shape	74 (11.43)	93.09 (1.14)	0.026	
Moderate-decrease	68 (10.51)	83.31 (1.43)	0.223	
High-increase	18 (2.78)	105.40 (9.40)	0.030	

Model was adjusted for age, sex, duration of diabetes, smoking, during, education, BMI, SBP, TC, TG, HDL, LDL and hypertension

### Subgroup analysis

Additional file 1: Table S1 showed the results of the subgroup analysis of baPWV in sex group. The P value for trend of ΔbaPWV on males and females were 0.004 and < 0.001, respectively. Although, the trend between HbA1c and baPWV still could be observed, the adjusted mean values of ΔbaPWV for the high-increase group for males and females were 56.39 (6.88) and 289.57 (8.59), respectively.

## Discussion

In this longitudinal cohort study, we descripted four trajectories’ groups of HbA1c (Low-stable, U-shape, Moderate-decrease, High-increase) during follow-up, which presented the dynamic changes of HbA1c in patients with diabetes during long-term treatment. We also explored the association between long-term glycemic control and the progression of vascular stiffness. These findings provided high-quality evidence of the causal relationship between diabetes and arterial stiffness on a time scale.

The latent class mixture model (LCMM) was one of the trajectories analysis methods, which was used to estimate the latent trajectories group of depressive symptoms in the early time [15, 18]. Compared with the traditional analysis method of longitudinal data, it better informs etiological associations by phenotypic analysis of certain “at risk” subpopulations [19]; and LCTM offers a public health strategy to identify early divergent adverse trajectories as potential intervention targets [17, 20]. In this study, we identified four discrete trajectory groups of HbA1c according to the BIC; they were low-stable, U-shape, Moderate-decrease, High-increase, respectively. The trajectory grouping is consistent with clinical experience: some patients who have good compliance with treatment may disengage from hyperglycemia status and control their blood glucose at a low level; the other part of patients failed to maintain the treatment after the blood sugar stabilized, resulting in the blood glucose increased again; In addition, over the past two years, due to the impact of the epidemic of COVID19, some patients may not receive follow-up according to the prescribed time, which may be one of the reasons for the continuous rise of HbA1c [21, 22]. In future studies, we will continue to follow up with the patients of the High-increase trajectory group to illustrate the impact of the COVID-19 epidemic on HbA1c.

A Mendelian randomization analysis of the Chinese population found that a genetically determined decrease in insulin secretion was associated with increased baPWV [23]. For people with long-term poor blood glucose control, baPWV decreases faster. Moreover, the other studies demonstrated a two-way causal relationship between glucose homeostasis and arterial stiffness [24]. The potential mechanisms between diabetes development and arterial stiffness may be understood as follows. Impaired endothelial function can cause dysfunction in capillary relaxation, constriction, or sparse distribution, which in turn causes arterial wall hardening. Arterial stiffness then could damage the capillary, resulting in a vicious cycle. Second, arterial stiffness could cause functional damage to low-resistance organs, such as the pancreas, liver, and brain [25, 26]. In future study, we will conduct some basic research to elucidate the mechanism of blood glucose control and arteriosclerosis.

This study used a prospective cohort study to verify the association between HbA1c and baPWV. Longitudinal data help elucidate the causal association between changes in HbA1c and arteriosclerosis. The trajectory groups of HbA1c show the control level of blood glucose in diabetes patients during long-term treatment, and the trajectory curve contains hidden information such as initial blood glucose level, variation trend, and variation degree. Using the trajectories modeling method, we could simplify the difficulty of fitting statistical models. In this study, we described the association between HbA1c and baPWV to illustrate the influence of blood glucose changes on the progression of arteriosclerosis during long-term hyperglycemic therapy. All of the ΔbaPWV in four trajectory groups were greater than 0 and the value of P for trend was < 0.05, which means that the atherosclerosis level of the participants had progressed in the follow-up and the progress rate of the 3rd and 4th trajectory groups is greater than the 1st and 2nd trajectory groups.

Although the main conclusion of this research was consistent between female and male (The P value for trend of ΔbaPWV on males and females were 0.004 and < 0.001, respectively.), the difference of mean ΔbaPWV between the male and female in high-growth groups should be considered (ΔbaPWV was 56.39 and 289.57, respectively). The following reasons may explain this phenomenon. Some studies reported that there was a substantial augmentation of the risk for arterial stiffness after menopause, resulting in a curve-linear increase of baPWV [27], which was consistent with Fig. 1 of this study. However, the female participants of this research concentrated among younger age groups (Additional file 1: Fig. S2). Even if our model adjusted the covariables such as age, the results show that differences in participant characteristics may not be balanced. On the other hand, due to the limitation of the study population, the sample size of this study is relatively small, especially in subgroup analysis. This problem could be also reflected in Additional file 1: Fig. S1, where the standard error in high and increase group was much higher than low-stable group.

In sensitivity analysis, the P for trend was 0.017 of ΔbaPWV in trajectory groups after excluding participants taking lipid-lowering medications. However, compared with results of overall, the ΔbaPWV was much smaller in each trajectory group of participants without taking lipid-lowering medications. One reason was that atherosclerosis progressed more slowly in people taking lipid-lowering drugs when their blood lipid levels were controlled. Yan et al. reported that people with relative lower lipid in life-course had a smaller subclinical atherosclerosis risk [28].

Major strengths of our study were that it was a national project to manage metabolic patients according to the same standard, as well rigorous and comprehensive measurements of risk factors were collected, which means that the model could adjust more potential confounding variables.

This study also has several limitations. First, the sample size was relatively insufficient for subgroup analysis, so we could not explain the stability of results in different characteristics of participants, e.g., hypoglycemic drugs and education level. Second, due to the prevalence of COVID-19, patients may decrease the time of follow-up visits by MMC. However, we could not evaluate how the trajectories of HbA1c were affected by COVID-19. Third, the median follow-up time of this study was 37.6 months; the conclusion may be more potent with the increase in follow-up time. Last, we could not include the patients with type 1 diabetes and gestational diabetes mellitus who have younger age and morbid states, which should be a reappraisal of the association between HbA1c and baPWV. Therefore, more natural population cohort studies should be conducted to elaborate on the two-way causal relationship.

## Conclusion

We found four different trajectories groups of HbA1c during the long-term treatment of diabetes. In addition, the result proves the causal relationship between long-term glycemic control and arterial stiffness on a time scale.

## Supplementary information

**Additional file 1: Figure S1. **Data cleaning procedure. **Figure S2. **Theage dependent trend of baPWV by sex. Age dependent of female. Agedependent of  male. The center lines arefitted by GAMLSS method, the other curves are probability density functions,and the horizontal axis represents the probability density for each age group. Thedensity of the data points is represented by the color chromaticity. **TableS1. **Covariate-adjusted means of change of bapwv by HbA1c Trajectory Group in sexgroups.

## Data Availability

Obtained with the approval of corresponding author.

## Abbreviations

SBP: Systolic blood pressure
HbA1c: Glycosylated hemoglobin
baPWV: Brachial-ankle pulse wave velocity
HDL: High density lipoprotein
LDL: Low density lipoprotein
TC: Total cholesterol
TG: Triglyceride
Glu: Fasting blood glucose
BMI: Body mass index
DN: Diabetic nephropathy
CVD: Cardiovascular disease
GAMs: The generalized additive models
