# Supplementary material for: Trajectories of glycated hemoglobin of T2DM and progress of arterial stiffness: a prospective study
Source: Diabetol Metab Syndr. 2023 Jun 23;15:135. doi: 10.1186/s13098-023-01108-8 (PMC10288736; doi:10.1186/s13098-023-01108-8)
Supplement: Supplementary file 1 — Additional file 1: Figure S1. Data cleaning procedure. Figure S2. Theage dependent trend of baPWV by sex. Age dependent of female. Agedependent of male. The center lines arefitted by GAMLSS method, the other curves are probability density functions,and the horizontal axis represents the probability density for each age group. Thedensity of the data points is represented by the color chromaticity. TableS1. Covariate-adjusted means of change of bapwv by HbA1c Trajectory Group in sexgroups. [file 13098_2023_1108_MOESM1_ESM.docx]

**Supplement**

**
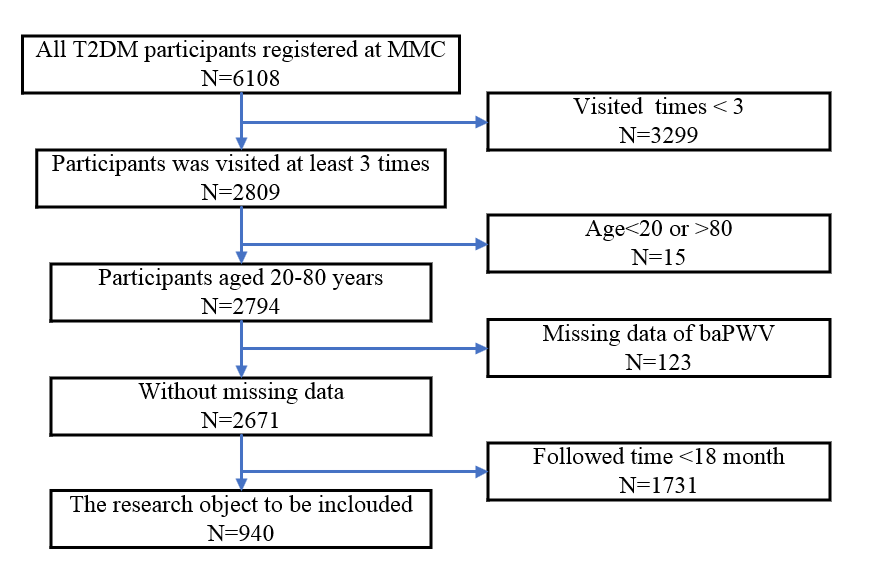
**

**Supplement Figure 1 Data cleaning procedure.**

**
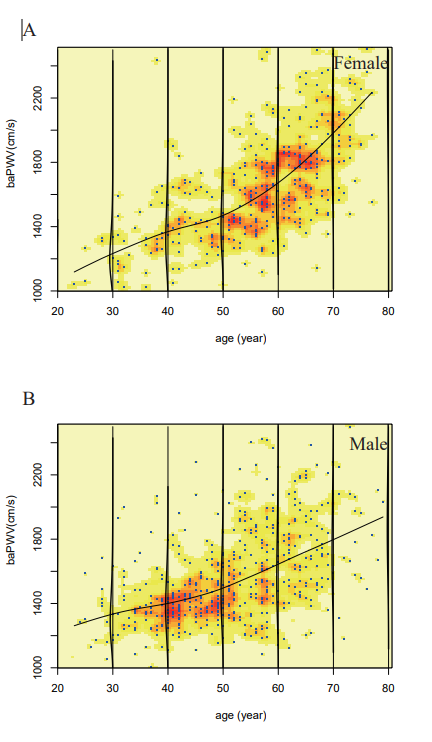
**

**Supplement Figure 2** The age dependent trend of baPWV by sex. (A). Age dependent of female. (B). Age dependent of male. The center lines are fitted by GAMLSS method, the other curves are probability density functions, and the horizontal axis represents the probability density for each age group. The density of the data points is represented by the color chromaticity.

**Supplemental Table 1 Covariate-Adjusted Means of change of bapwv by HbA1c Trajectory Group in sex groups**

| Trajectory group | Males | | |  | Females | | |  |
| --- | --- | --- | --- | --- | --- | --- | --- | --- |
|  | Participants, n(%) | Change of baPWV, mean(SE), cm/s | P value | P for trend | Participants, n(%) | Change of baPWV, mean(SE), cm/s | P value | P for trend |
| low−stable | 408 | 56.31 (0.12) | NA | 0.004 | 315 | 98.05 (0.19) | NA | <0.001 |
| U shape | 61 | 77.66 (1.71) | 0.091 |  | 27 | 129.83(5.25) | 0.175 |  |
| moderate decrease | 56 | 88.96 (1.71) | 0.025 |  | 42 | 152.05(1.94) | <0.001 |  |
| high and increase | 18 | 56.39 (6.88) | 0.513 |  | 13 | 289.57(8.59) | <0.001 |  |

Model was adjusted for age, duration of diabetes, smoking, during, education, BMI, SBP, TC, TG, HDL, LDL and hypertension.
